# Supplementary material for: Identification of photoperiod-induced specific miRNAs in the adrenal glands of Sunite sheep (Ovis aries)
Source: Front Vet Sci. 2022 Jul 22;9:888207. doi: 10.3389/fvets.2022.888207 (PMC9354845; doi:10.3389/fvets.2022.888207)
Supplement: Supplementary file 1 [file Data_Sheet_1.ZIP › Supplementary file/miRDeep2 file.zip!/miRDeep2 file/Know_Structure/LP42A2/oar-mir-191.pdf]

miRBase precursor	: oar-mir-191
Total read count	: 81430
oar-miR-191 read count	: 81199
remaining reads	: 231

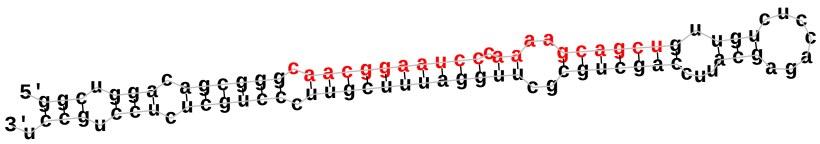

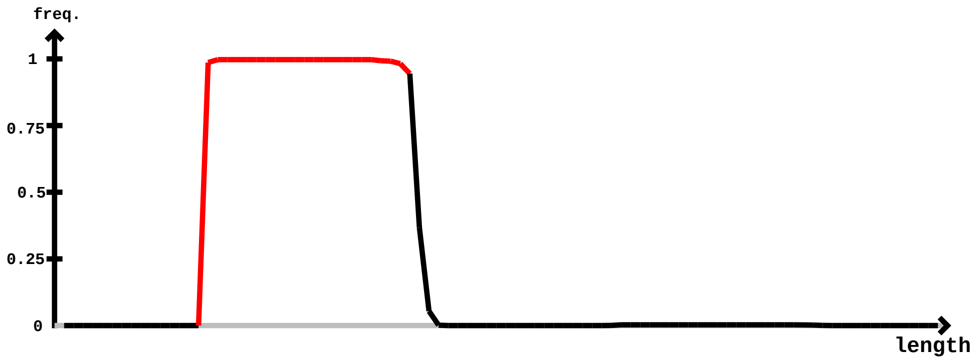


```
                oar-miR-191
5'- ggcuggacagcgggcaacggaaucccaaaagcagcuguugucuccagagcauuccagcugcgcuuggauuucguucccugcucuccugccu   -3'    exp
    (((.(((.((((((.((((((((((.((..(((((((.(((.......)))...)))))))..))))))))))))).)))))).))).))).    reads     mm         sample
    ..............caacggaaucccaaaag............................................................    3         0          seq
    ..............caacggaaucccaaGagc...........................................................    1         1          seq
    ..............caacggaaucccaaaagc...........................................................    347       0          seq
    ..............caacggaaucccaaaagca..........................................................    116       0          seq
    ..............caacggaaucccaaaagcag.........................................................    753       0          seq
    ..............caacggaaucccaaaagcagc........................................................    3037      0          seq
    ..............caacggaaucccaaaagcagcu.......................................................    46794     0          seq
    ..............caacggaaucccaaaagcagcug......................................................    25117     0          seq
    ..............caacggaaucccaaaagcagcugu.....................................................    4029      0          seq
    ..............caacggaaucccaaaagcagcuguu....................................................    113       0          seq
    ..............caacggaaucccaaaagcagcuguug...................................................    2         0          seq
    ...............aacggaaucccaaaagca..........................................................    1         0          seq
    ...............aacggaaucccaaaagcag.........................................................    3         0          seq
    ...............aacggaaucccaaaagcagc........................................................    11        0          seq
    ...............aacggaaucccaaaagcagcu.......................................................    232       0          seq
    ...............aacggaaucccaaaagcagcug......................................................    369       0          seq
    ...............aacggaaucccaaaagcagcugu.....................................................    254       0          seq
    ...............aacggaaucccaaaagcagcuguu....................................................    8         0          seq
    ...............aacggaaucccaaaagcagcuguug...................................................    1         0          seq
    ................acggaaucccaaaagcagcu.......................................................    3         0          seq
    ................acggaaucccaaaagcagcug......................................................    5         0          seq
    .......................................................agcugcgcuuggauuucguuc...............    3         0          seq
    ........................................................gcugcgcuuggauuucgu.................    1         0          seq
    ........................................................gcugcgcuuggauuucguu................    1         0          seq
    ........................................................gcugcgcuuggauuucguuc...............    10        0          seq
    ........................................................gcugcgcuuggauuucguucc..............    8         0          seq
    ........................................................gcugcgcuuggauuucguuccc.............    16        0          seq
    .........................................................cugcgcuuggauuucgu.................    1         0          seq
    .........................................................cugcgcuuggauuucguu................    6         0          seq
    .........................................................cugcgcuuggauuucguuc...............    16        0          seq
    .........................................................cugcgcuuggauuucguucc..............    32        0          seq
    .........................................................cugcgcuuggauuucguuccc.............    95        0          seq
    .........................................................cugcgcuuggauuucguucccu............    36        0          seq
    ..........................................................ugcgcuuggauuucguucc..............    1         0          seq
    ..........................................................ugcgcuuggauuucguucccu............    5         0          seq
```

oar-miR-191

ggcuggacagcgggcaacggaaucccaaaagcagcuguugucuccagagcauuccagcugcgcuuggauuucguucccugcucuccugccu
